# Post-Chemotherapy Canine Lymphomatous Lymph Node Observations on B-Mode and Strain Elastographic Ultrasound

**DOI:** 10.3390/vetsci11080352

**Published:** 2024-08-04

**Authors:** Somchin Sutthigran, Phasamon Saisawart, Suphat Soeratanapant, Patharakrit Teewasutrakul, Sirintra Sirivisoot, Chutimon Thanaboonnipat, Anudep Rungsipipat, Nan Choisunirachon

**Affiliations:** 1 Department of Surgery, Faculty of Veterinary Science, Chulalongkorn University, Bangkok 10330, Thailand; zomchin@mut.ac.th (S.S.); fvetbws@gmail.com (P.S.); dentpigmann@gmail.com (S.S.); chutimon.th@chula.ac.th (C.T.); 2 Small Animal Teaching Hospital, Faculty of Veterinary Science, Chulalongkorn University, Henri Dunant Rd., Bangkok 10330, Thailand; patharakrit@yahoo.com; 3 Center of Excellence for Companion Animal Cancer, Department of Veterinary Pathology, Faculty of Veterinary Science, Chulalongkorn University, Henri Dunant Rd., Bangkok 10330, Thailand; sirintra.s@chula.ac.th (S.S.); anudep.r@chula.ac.th (A.R.)

**Keywords:** B-mode, canine, elastography, multicentric lymphoma, ultrasound scores, ultrasound

## Abstract

**Simple Summary:**

Canine multicentric lymphoma is a common hematopoietic neoplasm in dogs that initially responds well to treatment but often relapses due to chemotherapy resistance. Evaluating treatment response is crucial. Ultrasound can differentiate between benign and malignant lymph nodes, but its use post chemotherapy is limited. This study compared ultrasound parameters of lymphomatous lymph nodes during the first three weeks post treatment with normal lymph nodes from healthy dogs. Ultrasound, including B-mode and elastography, was performed pre-treatment and weekly for three weeks post treatment. Lymphomatous lymph nodes were categorized into partial response and stable disease groups. Ultrasound scores, combining B-mode and elastography parameters, were significantly higher in lymphomatous lymph nodes than normal lymph nodes. Lymphomatous lymph nodes at pre-treatment had higher values than post-treatment. Ultrasound scores significantly differed among healthy, partial response, and stable disease groups. Therefore, ultrasound can be effectively used in conjunction with the conventional method to evaluate treatment response.

**Abstract:**

Canine multicentric lymphoma (CML) is a prevalent hematopoietic neoplasm that initially responds well to treatment but often relapses due to chemotherapy resistance. Evaluation of treatment response is essential for effective management. Ultrasound (US) can differentiate between benign and lymphomatous lymph nodes (LLNs). However, its utility in monitoring LLNs post chemotherapy is limited. This study aimed to compare US parameters of LLNs during the first 3 weeks post treatment and evaluate their diagnostic performance compared with the conventional method for assessing treatment response. This study included 95 LLNs from 15 dogs with CML and 60 normal lymph nodes (NLNs) from 15 healthy dogs. US, including B-mode and elastography, was performed pre-treatment and weekly for 3 weeks post treatment, and compared with the results of NLNs. LLNs were categorized into partial response and stable disease groups using the conventional method. US scores were established by combining B-mode and elastography parameters. The results showed significantly higher values of LLNs in the short-to-long axis ratio, elastographic scales, and blue-to-green color histogram compared with NLNs. Additionally, LLNs at pre-treatment had significantly higher values than LLNs post treatment. US scores significantly differed among the healthy, partial response, and stable disease groups. In conclusion, B-mode US, elastography, and US scores demonstrated changes during chemotherapy consistent with the conventional method and can be used in conjunction with the conventional method to evaluate the treatment response of CML.

## 1. Introduction

Lymphoma is a prevalent hematopoietic neoplasm in dogs, constituting 83% of all canine hematopoietic neoplasms [1]. Among these, canine multicentric lymphoma (CML), characterized by multiple peripheral lymphadenopathies, accounts for >80% of all canine lymphomas [1]. Chemotherapy is the treatment of choice for CML in dogs, exhibiting a high initial response rate. However, tumor relapse due to treatment resistance remains a challenge [2,3]. The evaluation of tumor response to chemotherapy is critical for successful treatment and aids veterinarians in treatment planning and adjusting appropriate chemotherapeutic protocols for long-term management. Currently, the standardized method for evaluating treatment responses based on consensus from the veterinary cooperative oncology group (VCOG) [4], involves the measurement of peripheral lymph nodes. However, this method can be highly subjective because it relies on the sole detection of gross alterations in peripheral lymph nodes accessible or palpable through the skin [5].

Ultrasound (US) is a non-invasive modality facilitating the visualization and evaluation of lymph node characteristics [6,7,8]. In veterinary medicine, B-mode US is useful in distinguishing between benign and malignant lymph nodes [6,8]. Malignant lymph nodes tend to be characterized by a heterogeneous echotexture, absence of a hyperechoic hilum, an increased short-to-long axis (S/L) ratio exceeding 0.5, and hyperechoic perinodal fat [6,8]. However, B-mode US images alone are insufficient for accurate differentiation between malignant and benign lymph nodes, particularly concerning inflammatory lymph nodes [9,10]. Doppler color flow and power Doppler US have also been used to differentiate between benign and malignant lymph nodes. Malignant lymph nodes tend to have mixed vascular distribution, higher resistivity, and pulsatility indices [6,11]. Additionally, contrast-enhanced ultrasound has been reported to identify lymphomatous nodes in dogs by detecting malignant vascular characteristics [12]. Strain elastography (SE) enables the assessment of tissue stiffness alterations caused by specific pathological or physiological processes [13]. Malignant lymph nodes tend to exhibit alterations in intranodal structures and increased tissue stiffness [8,14]. Consequently, SE parameters such as elastographic scales (ES) and image color histograms can be useful in distinguishing between benign and malignant lymph nodes, including lymphomatous lymph nodes (LLNs) [6,7,8]. However, the use of either B-mode US or SE may not consistently provide accurate results in differentiating between benign and malignant lymph nodes [7,15]. Combining both techniques may enhance diagnostic precision [7].

Several veterinary studies have explored the use of US to differentiate between benign and malignant nodes including lymphomas [6,7,8,15,16], limited information exists on its application, particularly the combination of B-mode US and SE, in evaluating chemotherapy response and monitoring CML. Therefore, this study aims (1) to compare the parameters of the S/L ratio and B-mode appearances, including ES and the image color histogram, between normal lymph nodes (NLNs) and LLNs; (2) to assess weekly changes in the US parameters of LLNs during the first cycle of chemotherapy; and (3) to establish a US scoring system using a combination of B-mode US and SE parameters to evaluate LLNs during chemotherapy and compare this outcome with those of the conventional standardized method for evaluating treatment response.

## 2. Materials and Methods

### 2.1. Animals

This research was conducted as a prospective observational study. Thirty client-owned dogs that presented to the Small Animal Hospital, Faculty of Veterinary Science, Chulalongkorn University between January 2022 and December 2023 were included in the study. Of these 15 dogs were diagnosed with CML (CML group), and 15 healthy dogs were included in the healthy group. Inclusion criteria for the CML group were as follows: (i) dogs presenting with peripheral lymphadenopathy and (ii) dogs diagnosed with CML by cytological or histological examinations and/or including immunohistochemistry or immunocytochemistry. Additionally, a cytological examination of the liver and spleen, as well as a peripheral blood smear, was performed. Substaging was determined based on the presence or absence of systemic signs. Data were recorded to establish clinical staging based on the WHO staging system [17]. CML dogs were treated with either the combination of cyclophosphamide (250 mg/m^2^ orally divided over 2 consecutive days in the second week), vincristine (0.7 mg/m^2^ intravenous injection in the first week), and prednisolone (2 mg/kg orally daily at the first week, tapering over 4 weeks to 0.5 mg/kg orally daily) (COP) or the combination of COP with L-asparaginase (400 units/kg subcutaneous injection once in the first week) (L-COP). CML dogs with a history of previous treatment with chemotherapy or steroid treatments were excluded. Inclusion criteria for dogs in the healthy group were as follows: (i) dogs with no abnormalities detected during physical examination, including NLNs upon palpation, and (ii) dogs with blood parameters within normal limits. Dogs with clinical signs of any disease or those that had received prior steroid treatment were excluded. Clinical information, such as breed, sex (including neuter status), age, and body weight (BW), was recorded for all enrolled dogs.

### 2.2. Analysis of Ultrasonographic Images

**Figure 1:**
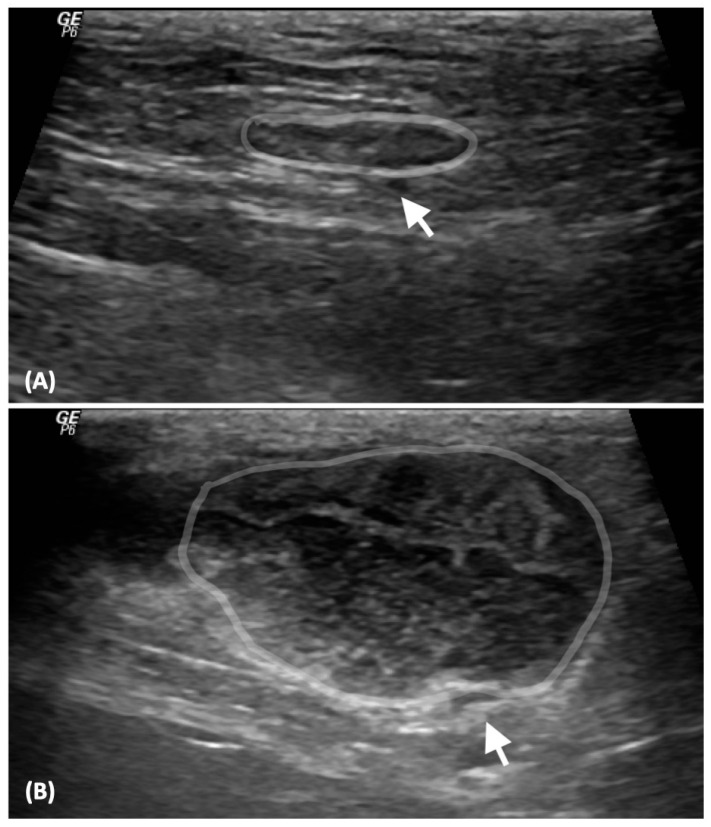
B-mode ultrasonographic images of a normal lymph node (gray line, (**A**)) and a lymphomatous node (gray line, (**B**)). A normal lymph node exhibits a regular contour, well-defined margin, homogeneous echotexture, presence of a nodal hilum and the isoechoic perinodal fat appears (arrow, (**A**)). A lymphomatous node displays an irregular contour, well-defined margin, heterogeneous echotexture, absence of a nodal hilum and the hyperechoic perinodal fat becomes (arrow, (**B**)).

US examinations were conducted on the affected lymph nodes of LLNs including the popliteal, inguinal, prescapular, and medial iliac lymph nodes, weekly from the first presentation to the third week post chemotherapy. LLNs were categorized as LLN0 at pre-treatment, LLN1 at the first week post treatment, LLN2 at the second week post-treatment, and LLN3 at the third week post treatment. Additionally, US examinations of the inguinal and medial iliac lymph nodes were performed for NLNs at the first presentation. An experienced diplomate from the Thai Board of Veterinary Surgery and a radiologist conducted all lymph node examinations using a high-frequency linear transducer (10 MHz) (Logiq P6 GE Healthcare, Japan). The B-mode US images for NLN and LLN were retrieved and recorded once. The following B-mode US parameters were measured and recorded: (i) maximum long axis (L) and maximum short axis (S) on the sagittal plane, (ii) S/L ratio, and (iii) lymph node appearance characteristics, including contour regularity (regular—smooth outline or irregular—uneven outline), nodal border definition (well-defined margin—clearly distinguished from surrounding tissue or ill-defined margin—poorly distinguished from surrounding tissue), parenchymal uniformity (homogeneous echotexture or heterogeneous echotexture), nodal hilum (presence or absence of a hyperechoic center), and perinodal fat echogenicity compared with the lymph node (isoechoic—similar echogenicity or hyperechoic—increased echogenicity) (Figure 1).

**Figure 2:**
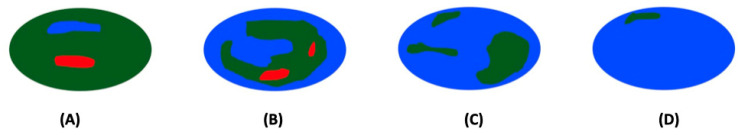
Elastographic scales of lymph nodes [16] (**A**–**D**). Scale 1 as soft (predominantly red with less than 10% of the blue area (**A**); Scale 2 as moderately soft (predominantly red and green with 10–50% of the blue area) (**B**); Scale 3 as moderately stiff (predominantly blue and green with 50–90% of the blue area) (**C**); and Scale 4 as stiff (predominantly blue more than 90%) (**D**).

SE was performed immediately after B-mode US using the same US machine and transducer. A high-frequency linear transducer (10 MHz) was used, and noise rejection along with enhanced image smoothing was activated by the US machine software (Logiq P6 application software R1.0.4) to obtain high-quality images for analysis. The transducer was gently applied to the skin over the lymph node and rhythmic compression and decompression were performed perpendicular to the lymph nodes. Data were collected when a quality bar showed the maximum green color indicating signal regularity and conformity. SE images were captured and recorded three times, analyzed both qualitatively and quantitatively, and the average value was calculated for analysis. For qualitative analysis, ES was determined and recorded according to a previous method [16]: (i) scale 1 as soft (predominantly red with <10% of the blue area), (ii) scale 2 as moderately soft (predominantly red and green with 10–50% of the blue area), (iii) scale 3 as moderately stiff (predominantly blue and green with 50–90% of the blue area), and (iv) scale 4 as stiff (predominantly blue with >90%) (Figure 2A–D).

**Figure 3:**
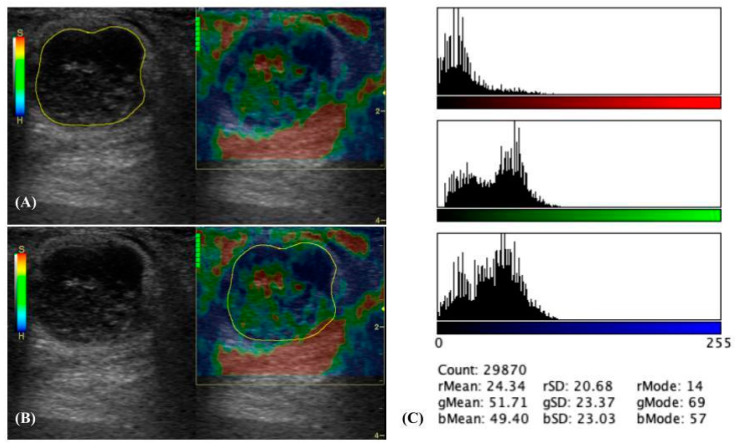
Image color histogram analysis of lymphomatous node (**A**–**C**). First, the region of interest (ROI) is manually drawn covering the entire lymph node within its internal border, excluding perinodal fat on the B-mode image (**A**). The same ROI will be inserted into the strain elastography image (**B**). The image color histogram values of blue, green, and red colors were analyzed by Image J software.

For the quantitative analysis, the image color histogram was evaluated using Image J software version 1.54j (U. S. National Institutes of Health, Bethesda, MD, USA) and its software plug-in for image color histograms. Initially, the region of interest (ROI) was manually drawn on the B-mode image, encompassing the entire lymph node within its internal border, excluding perinodal fat. This ROI was used to evaluate the complete image color histogram of the lymph node. Subsequently, the same ROI was applied to the SE images. The average image color histogram values from three images of blue, green, and red colors were then constructed and recorded. Additionally, the ratio of the image color histogram between blue and green colors (B/G ratio) was calculated and recorded (Figure 3A–C).

Receiver operating characteristics (ROC) curve and area under the curve (AUC) were generated to determine the optimal cut-off value of US parameters including S/L ratio, ES, image color histogram values of blue green, and red colors as well as B/G ratio, to differentiate between NLNs and LLNs.

US scores were established to differentiate between NLNs and LLNs including pre-treatment LLNs and those at post-treatment. These scores were combined using both B-mode US and SE information. Eight criteria were used, as listed in Table 1.

Furthermore, the US scores were compared with those of the conventional standardized method for evaluating treatment response. LLN responses to chemotherapy were evaluated following the response evaluation criteria outlined in the VCOG guidelines [4] which classified responses as either partial response (at least a 30% decrease in the longest diameter) or stable disease (neither a sufficient decrease for partial response nor a sufficient increase for progressive disease). These categories were then compared with the US scores.

### 2.3. Statistical Analysis

Statistical analysis was performed by using a commercial software program (SPSS Statistics software for MacOS version 29, IBM, IBM Corp., New York, NY, USA). Descriptive analyses were used to present all clinical data of the enrolled dogs. The normal distribution of continuous data was assessed using the Shapiro–Wilk normality test. Normally distributed data such as age, BW, S/L ratio, and image color histogram were presented as mean ± standard deviation (SD), while non-normally distributed data such as ES were presented as median and range. Age and body weight were compared between the healthy and lymphoma groups using an independent samples t-test. Sex was compared using a chi-square test. Quantitative data, including US score, S/L ratio, and image color histogram, were compared among LLN0-LL3 groups using Friedman’s Two-way Analysis of Variance by Ranks and between the NLN and LLN0–LLN3 groups using the Kruskal–Wallis test. Moreover, the US scores were compared among the NLN, partial response LLN, and stable disease LLN groups based on the VCOG guidelines using the Kruskal–Wallis test. Statistical analyses will be considered statistically significant if *p*-values are less than 0.05.

## 3. Results

### 3.1. Clinical Demographic Data

Among the 15 dogs in the CML group, there were mixed-breed dogs (n = 4), Chihuahua (n = 3), Beagle (n = 2), and Jack Russell (n = 2), with one each of the Cavalier King Charles Spaniel, French Bulldog, Shih Tzu, and Welsh Corgi. All dogs in the CML group were classified as having high-grade lymphoma according to the Kiel Cytological Classification. Additionally, 6 dogs (46.67%) were diagnosed with B-cell lymphoma via immunocytochemistry, and 1 dog was diagnosed with diffuse large B-cell lymphoma through immunohistopathological examination. Of the 15 CML dogs, 12 (80%) were treated using the L-COP protocol, while 3 (20%) dogs were treated with the COP protocol. In the healthy group, the dogs included mixed-breed dogs (n = 4), Chihuahua (n = 3), Shih Tzu (n = 2), Poodle (n = 2), and one each of the Bichon Frise, German Shepherd, Miniature Pinscher, and Siberian Husky breeds. There was no significant difference in age and body weight between the two groups. However, there was a significant difference in sex between the groups. The clinical demographic data of all enrolled dogs are presented in Table 2. The number and frequency of WHO stages and substages, along with the treatment protocols in the canine multicentric lymphoma (CML) group, are presented in Table 3.

A total of 95 LLNs were collected from 15 dogs with CML and a total of 60 NLNs from 15 healthy dogs were included in this study. The distribution, number, and frequency of the enrolled lymph nodes are shown in Table 4.

### 3.2. Comparison of US Parameters between NLNs and LLNs

From these 30 dogs, 60 NLNs were obtained from 15 healthy dogs, and 53 LLNs were obtained from 15 CML dogs. For the LLNs, all 53 LLNs were performed US and recorded at pre-treatment (LLN0), the first week post treatment (LLN1), the second week post treatment (LLN2), and the third week post treatment (LLN3). All US parameters were compared between NLNs and LLN0-3 among comparable lymph node locations, including the inguinal and medial iliac lymph nodes are presented in Table 5. These parameters include the S/L ratio, US appearances, ES, and color histogram (red, blue, green, and B/G ratio).

**Figure 4:**
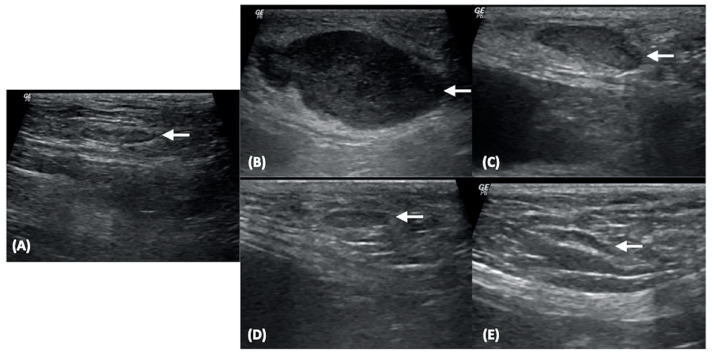
B-mode ultrasonographic images of normal superficial inguinal lymph node (arrow, **A**), lymphomatous node at the pre-treatment (arrow, **B**), lymphomatous node at 1 week post treatment (arrow, **C**), lymphomatous node at 2 weeks post treatment (arrow, **D**), and lymphomatous node at 3 weeks post treatment (arrow, **E**).

**Figure 5:**
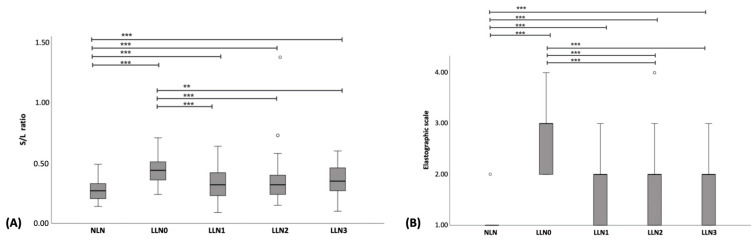
Boxplot of the short-to-length (S/L) axis ratio (**A**) and elastographic scales (**B**) for normal lymph node (NLN), lymphomatous node at the pre-treatment (LLN0), lymphomatous node at 1 week post-treatment (LLN1), lymphomatous node at 2 weeks post treatment (LLN2), and lymphomatous node at 3 weeks post treatment (LLN3). The Kruskal–Wallis test was used to compare NLN with LLN0-LLN3. ** = *p* < 0.01; *** = *p* < 0.001.

On B-mode US, the S/L ratio of NLNs was significantly lower than that of LLN0–LLN3 (*p* < 0.001). Additionally, the S/L ratio of LLN0 was significantly higher than those of LLN1 (*p* < 0.001), LNN2 (*p* < 0.001), and LNN3 (*p* = 0.002) (Figure 4A–E). However, no significant differences in S/L ratios were found among LLN1–LLN3. The boxplot of the S/L ratios of NLNs and LNN0–LLN3 at the inguinal and medial iliac lymph nodes is shown in Figure 5A.

**Figure 6:**
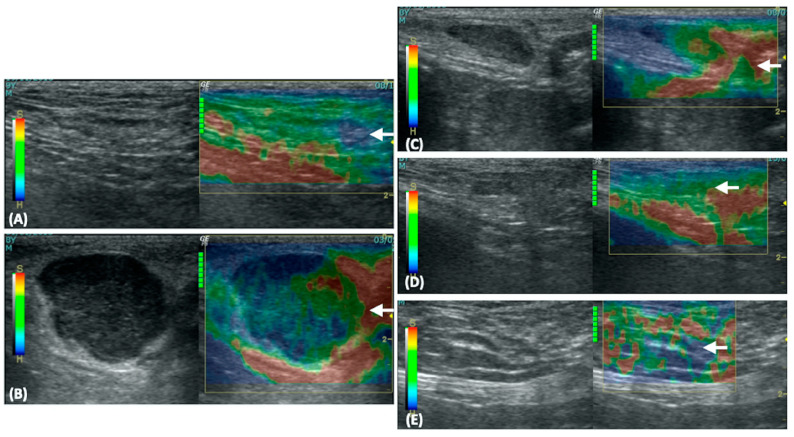
Strain elastographic images of normal lymph node with elastographic scale (ES) of 1 (arrow, **A**), lymphomatous node at the pre-treatment with ES of 3 (arrow, **B**), lymphomatous node at 1 week post treatment with ES of 1 (arrow, **C**), lymphomatous node at 2 weeks post treatment with ES of 1 (arrow, **D**), and lymphomatous node at 3 weeks post treatment with ES of 1 (arrow, **E**).

**Figure 7:**
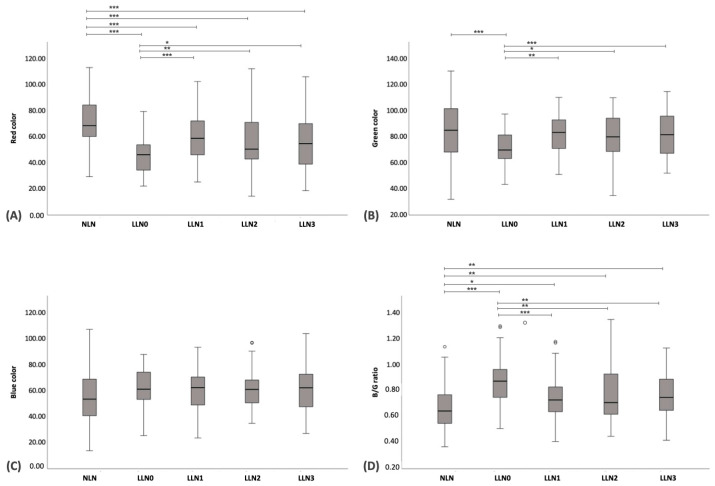
Boxplots of the color histogram value for normal lymph node (NLN), lymphomatous node at the pre-treatment (LLN0), lymphomatous node at 1 week post treatment (LLN1), lymphomatous node at 2 weeks post treatment (LLN2), and lymphomatous node at 3 weeks post treatment (LLN3). The Kruskal–Wallis test was used to compare NLN with LLN0-LLN3. The boxplots represent the values for red color (**A**), green color (**B**), blue color (**C**), and blue-to-green (B/G) ratio (**D**). * = *p* < 0.05; ** = *p* < 0.01; *** = *p* < 0.001.

Regarding SE, the ES value of NLNs was significantly lower than that of LLN0–LLN3 (*p* < 0.001). Furthermore, the ES value of LLN0 was significantly higher than those of LLN1–LLN3 (*p* < 0.001). No significant differences in ES values were found among LLN1–LLN3 (Figure 6A–E). The boxplot of the ES of NLNs and LNN0–LLN3 at the inguinal and medial iliac lymph nodes is shown in Figure 5B. Regarding the color histogram, the red color histogram value of NLN was significantly lower than that of LLN0 (*p* < 0.001) but was significantly higher than those of LLN1 (*p* = 0.005), LLN2 (*p* < 0.001), and LLN3 (*p* < 0.001). Additionally, the red color histogram value of LLN0 was significantly higher than those of LLN1 (*p* < 0.001), LLN2 (*p* = 0.003), and LLN3 (*p* = 0.003). Furthermore, the green color histogram value of NLNs was significantly higher than that of LLN0 (*p* < 0.001), but no significant difference was observed among LLN1–LLN3. Moreover, the green color histogram value of LLN0 was significantly lower than those of LLN1 (*p* = 0.003), LLN2 (*p* = 0.005), and LLN3 (*p* = 0.002). However, there were no significant differences in the blue color histogram between NLNs and each of LLN0–LLN3. Regarding the B/G ratio, the B/G ratio value of NLNs was significantly lower than those of LLN0 (*p* < 0.001), LLN1 (*p* = 0.021), LLN2 (*p* = 0.034), and LLN3 (*p* = 0.003). Additionally, the B/G ratio value of LLN0 was significantly higher than those of LLN1 (*p* < 0.001), LLN2 (*p* < 0.003), and LLN3 (*p* = 0.003). However, there were no significant differences in the B/G ratio values among LLN1–LLN3. The boxplots of color histograms of red, green, blue, and B/G ratios in NLNs and LNN0–LLN3 at the inguinal and medial iliac lymph nodes are shown in Figure 7A–D.

### 3.3. Comparison of US Parameters among LLN at Different Time Points of Chemotherapy

**Figure 8:**
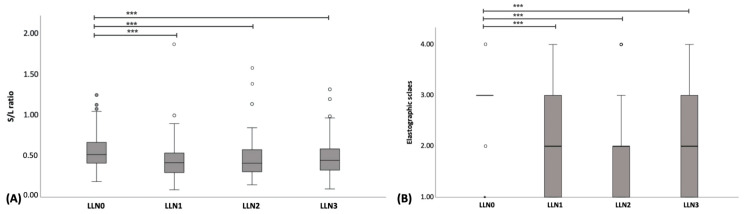
Boxplot of the short-to-length axis (S/L) ratio (**A**) and elastographic scales (**B**) for lymphomatous node at the pre-treatment (LLN0), lymphomatous node at 1 week post treatment (LLN1), lymphomatous node at 2 weeks post treatment (LLN2), and lymphomatous node at 3 weeks post treatment (LLN3). Friedman’s Two-Way Analysis of Variance by Ranks was used to compare the data among LLN0-LLN3. *** = *p* < 0.001.

In total, 95 LLNs from 15 CML dogs were compared based on B-mode US. The results revealed that the S/L ratio of LLN0 was significantly higher than that of LLN1 (*p* < 0.001), LLN2 (*p* < 0.001), and LLN3 (*p* < 0.001). However, no significant differences were observed among LLN1–LLN3. The boxplot of S/L ratios in the LLN0 –LLN3 is shown in Figure 8A.

**Figure 9:**
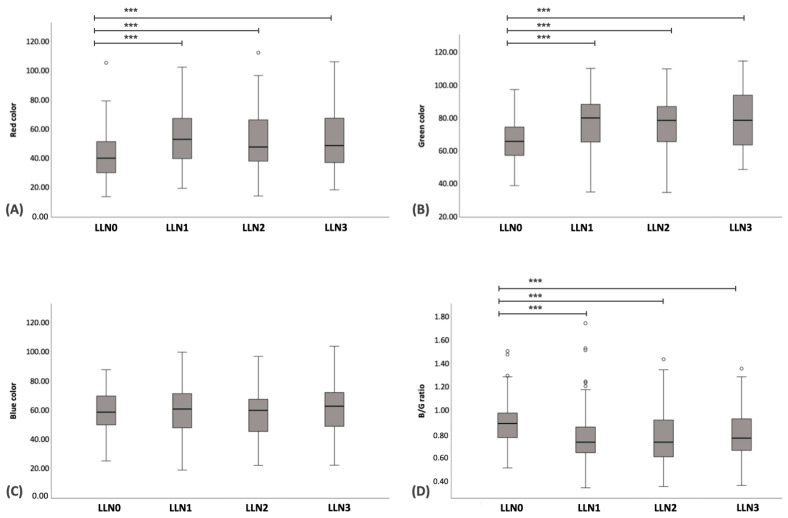
Boxplots of the color histogram value for lymphomatous node at the pre-treatment (LLN0), lymphomatous node at 1 week post treatment (LLN1), lymphomatous node at 2 weeks post treatment (LLN2), and lymphomatous node at 3 weeks post treatment (LLN3). Friedman’s Two-Way Analysis of Variance by Ranks was used to compare the data among LLN0-LLN3. The boxplots represent the values for red color (**A**), green color (**B**), blue color (**C**), and blue-to-green (B/G) ratio (**D**). *** = *p* < 0.001.

A total of 95 LLNs underwent SE, including medial iliac lymph nodes (n = 27), popliteal lymph nodes (n = 26), superficial inguinal lymph nodes (n = 26), and prescapular lymph nodes (n = 16). The ES of LLN0 was significantly higher than that of LLN1–LLN3 (*p* < 0.001); however, there were no significant differences observed among LLN1–LLN3 (Figure 6B–E). The boxplot of the ES in LLN0–LLN3 is shown in Figure 8B. Regarding the image color histogram, the red color histogram value of LLN0 was significantly lower than that of LLN1–LLN3 (*p* < 0.001). Similarly, the green color histogram value of LLN0 was significantly lower than that of LLN1–LLN3 (*p* < 0.001). However, there were no significant differences in the blue color histogram values among LLN0–LLN3. Finally, the B/G ratio value of LLN0 was significantly higher than that of LLN1–LLN3 (*p* < 0.001). No significant differences were observed in the B/G ratios among the LLN1–LLN3. The boxplot of the color histogram in LLN0–LLN3 is shown in Figure 9A–D, and all of the US parameters in LLN0–LLN3 are shown in Table 6.

### 3.4. Determination of Optimal Cut-Off Values of US Parameters between NLNs and LLNs

**Figure 10:**
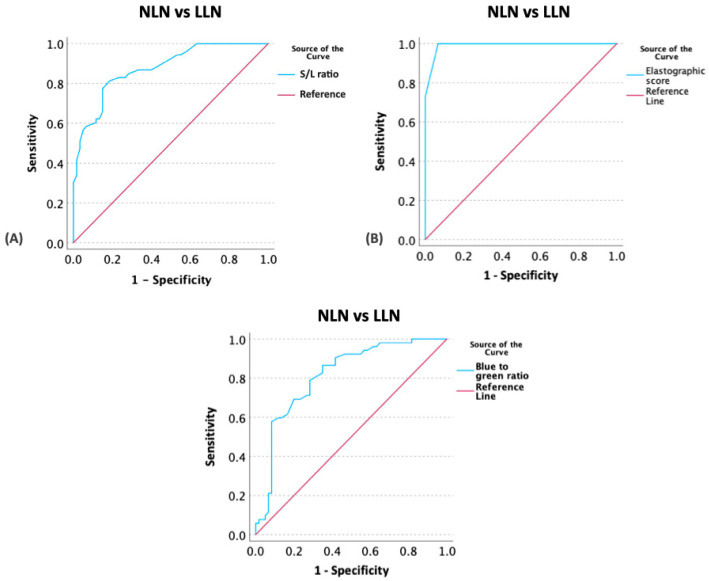
Receiver-operating characteristic curves assessing the cutoff value of the short-to-length axis (S/L) ratio (**A**), elastographic scales (**B**), and blue-to-green ratio (**C**) to distinguish between normal lymph nodes (NLNs) and lymphomatous nodes (LLNs).

ROC curves and AUC analysis were used to determine the optimal cut-off values for US parameters, including the S/L ratio, ES, and color histogram (red, green, blue, and B/G ratio), aimed at distinguishing between NLNs and LLNs. The optimal cutoff values for identifying lymphomatous nodes were determined to be greater than 0.345 for the S/L ratio (with 81.11% sensitivity; 95%CI 66.08–88.93% and 81.17% specificity; 95%CI 70.58–91.15%), 1.5 for the ES (with 100% sensitivity; CI 86.62–100% and 93.33% specificity; CI 89.86–99.91%), and 0.745 for the B/G ratio (with 80.80% sensitivity; 95%CI 57.09–81.54% and 71.70% specificity; 95%CI 64.64–87.75%), respectively (Figure 10; Table 7).

However, due to their loss of significance in the ROC and AUC analysis, no cut-off values could be established for the red and green color histograms.

### 3.5. Comparison of US Scores between NLNs and LLNs

**Figure 11:**
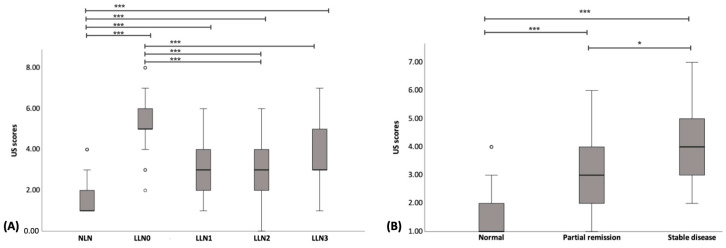
Boxplot of the ultrasonographic scores for normal lymph node (NLN), lymphomatous node at the pre-treatment (LLN0), lymphomatous node at 1 week post treatment (LLN1), lymphomatous node at 2 weeks post treatment (LLN2), and lymphomatous node at 3 weeks post treatment (LLN3) (**A**) and for the normal, partial remission, and stable disease groups (**B**). * = *p* < 0.05; *** = *p* < 0.001.

The median and range of US scores for NLNs were 1 (1–4). Whereas for LLN0, LLN1, LLN2, and LLN3, they were 5 (2–8), 3 (1–6), 3 (1–6), and 3 (1–7), respectively. Furthermore, the US scores of NLNs were significantly lower than those of LLN0–LLN3 (*p* < 0.001), whereas the US scores of LLN0 were significantly higher than those of LLN1–LLN3 (*p* < 0.001). The boxplot of US scores of NLNs and LLNs is shown in Figure 11A.

### 3.6. Comparison of US Scores with Conventional Treatment Response Evaluation

A total of 53 LLNs and 60 NLNs undergoing B-mode and SE were included for the comparison of US scores with the conventional treatment response evaluation method. Of the 53 LLNs, 24 (45.28%) were classified as stable diseases, while 29 (54.72%) were classified as partial remission. The US scores of NLNs were significantly lower than those of the partial remission (*p* < 0.001), and the stable disease (*p* < 0.001) groups. Moreover, the US scores of the partial remission group were significantly lower than those of the stable disease group (*p* = 0.041). The median and range of US scores were 1 (1–4) for NLNs, 3 (1–6) for LLNs with partial remission, and 4 (2–7) for LLNs with stable disease. The boxplot depicting US scores of NLNs and LLNs with partial remission and stable disease is displayed in Figure 11B.

## 4. Discussion

Our study found several key findings. US parameters such as S/L ratio, ES, and color histogram values (red, green, blue, and B/G ratio) significantly differed between NLNs and LLNs in dogs with CML. This suggests that the US may be a potential diagnostic tool for distinguishing between benign and malignant lymph nodes. Further, US scores, combining both B-mode and SE, showed significant differences between NLNs and LLNs, suggesting that this scoring system may be used in evaluating treatment response and monitoring disease progression in dogs with CML.

Using B-mode US, the S/L ratio has been proven to differentiate between NLNs and malignant lymph nodes in human and veterinary medicine [6,8,9,18,19]. Our study found a cut-off value of the S/L ratio for LLNs as ≥0.345. This value showed a high sensitivity of 81.11% and specificity of 81.17% and is lower than that reported in previous studies. Previous studies have reported a cutoff value of the S/L ratio greater than 0.5 for canine malignant lymph nodes [3,6,8]. Similar to findings in humans, malignant lymph nodes tend to have an S/L ratio exceeding 0.5 [18]. This suggests that malignant lymph nodes often have a round morphology due to tumor cell infiltration in a portion of the node. This obliterates lymphatic channels, leading to focal cortical enlargement [6]. The discrepancy in the cutoff values for the S/L ratio between studies can be attributed to several reasons. First, the current study primarily focused on LLNs, whereas previous studies included malignant lymph nodes with metastasis from various sources, such as mammary gland carcinoma or other carcinomas. This variation may have influenced the heterogeneous lymph node characteristics [6,9,19]. Second, the enrolled lymph nodes used to calculate the cutoff value in this study were collected from the superficial inguinal and medial iliac lymph nodes. Typically, these lymph nodes tend to exhibit a fusiform shape and an S/L ratio of <0.5 [20]. Our study findings indicate that despite LLN enlargement, they retained their fusiform shape and the S/L ratio of <0.5. Additionally, this study found a significant difference in the S/L ratio between LL0 and LL1–LLN3 during chemotherapy with COP or L-COP protocols. However, the S/L ratio did not return to normal when compared with that of NLNs. Regarding B-mode appearance, LLNs tended to have irregular contours, heterogeneous parenchyma, and hyperechoic perinodal fat. These findings are consistent with information from previous studies [6,21]. These appearances are more likely associated with infiltration of round-cell neoplasms including lymphoma [21]. Regarding nodal border definition, almost all lymphomatous nodes (>90%) in this study had well-defined margins. This finding is consistent with previous studies, which reported that approximately 60–80% of malignant lymph nodes had well-defined margins [6,22]. This is suspected to be because lymphomatous nodes tend to be more hypoechoic [22]. Interestingly, our study showed heterogeneous echotexture in 10 (16.77%) NLNs in healthy dogs. Although heterogeneous lymph nodes often suggest malignancy, previous studies have shown that they can also be observed in reactive lymph nodes. Reactive changes such as neutrophilic inflammation, lymphoid hyperplasia, and edema with hemosiderin can contribute to this heterogeneity [23]. Moreover, the mean age of the healthy group in our study was 10.86 years. Degenerative changes associated with aging, such as increased lipomatous atrophy, calcifications, and framework and capsular fibrosis, can significantly affect the internal structure of lymph nodes [24,25]. These age-related changes may lead to altered echotexture on ultrasound, contributing to the heterogenous lymph node. The nodal hilus is the natural entry point for blood vessels into the lymph node. It appears as a thin, central hyperechoic region and can be seen in both healthy and abnormal lymph nodes [26]. A previous study showed the presence of a nodal hilus in 75% of normal lymph nodes [22]. Conversely, our study found that 59 (98.33%) NLNs lacked a nodal hilus. This phenomenon is suspected to be caused by age-related degenerative changes or reactive changes that alter the internal structure of the lymph nodes [23,24,25]. Considering these factors is crucial, particularly in aging dogs, when interpreting ultrasonographic findings, as a heterogeneous echotexture and the absence of a nodal hilum do not necessarily indicate malignancy [6,26,27]. Additionally, previous studies have demonstrated that B-mode ultrasound parameters alone are insufficient for identifying malignant lymph nodes [7,19].

Malignant lymph nodes undergo intranodal structural changes, leading to alterations in tissue elasticity due to tumor cell growth, tumor angiogenesis, and fibrosis [6,14,15,28]. Tissue elasticity refers to its ability to regain its original size and shape after deformational force or stress. Tissue elasticity is inversely related to its stiffness or hardness and differences among tissue types are important for clinical diagnosis [29]. US elastography, a newer non-invasive imaging technique, can be used to assess tissue elasticity. It has been widely applied in veterinary medicine to investigate elasticity in various diseases, such as hepatic fibrosis [30], hepatic tumors [31], chronic kidney disease [32], mammary gland tumors [33], malignant lymph nodes [6,7,8,9], skin nodule such as mast cell tumor [34], and injured tendon [35]. In SE, stress force is applied to tissues causing internal displacements related to their elastic characteristics [28]. SE is a technique used for the semi-quantitative evaluation of tissue elasticity. This involves measuring the tissue deformation caused by manual compression and subsequent release. The degree of tissue deformation, compared to adjacent tissue, is visually represented as a color-coded map overlaid on B-mode US images. The results are displayed using a color scale, with blue color indicating regions of the low strain (hard area) and red indicating the regions of high strain (soft area) [8,28]. Using SE, ES is a method that evaluates tissue elasticity by assessing color-change lesions on a 4-point scale [16]. Malignant lymph nodes typically exhibit higher ES, indicating greater stiffness, compared with the benign lymph nodes [6,7,8,16]. Although ES can be used to predict malignant lymph nodes with a high sensitivity and specificity of 80% and 75%, it alone cannot distinguish between LLNs and metastatic nodes [6,15]. Our study confirmed higher ES values (ES = 3) in LLNs compared with NLNs (ES = 1), consistent with previous findings [6,7,15]. However, ES values overlap between benign and malignant lymph nodes, limiting its use as the sole parameter to evaluate malignancy [6,7,15]. In addition to ES, strain ratio is a pseudo-quantitative method, that compares strain in the ROI and that in the normal adjacent region, such as muscle or subcutaneous fat. Previous studies showed that a strain ratio > 1 indicates greater stiffness than the normal reference region [28] with a ratio > 1.5 indicating malignant lymph nodes [8]. However, the fixed ROI that cannot cover the entire lymph node and the difficult ROI selection in heterogeneous lymph nodes caused result discrepancy [36]. Conversely, strain histogram, another SE method, quantitatively evaluates tissue stiffness using Image J software for color histogram analysis. To analyze the elastographic images, ROI is drawn over the entire lymph node area. The pixel values within the ROI are then calculated and presented as red-green-blue (RGB) histogram values [8,31]. Malignant tissues typically show increased blue histogram values, while benign tissues usually exhibit higher red and green histogram values, reflecting their stiffness [31]. Our study found significantly higher red and green histogram values in NLNs compared with LLNs. However, no significant difference was observed in the blue histogram values between the two groups, possibly due to lymph node size variations affecting the ROI, subsequently leading to variations in the color histogram values. Conversely, the B/G ratio, indicating an increase in tissue stiffness due to a change in the proportion of blue and green color, was significantly higher in LLNs than in NLNs, consistent with a previous study [31].

The S/L ratio, ES, and image color histogram can differentiate between benign and malignant lymph nodes. However, relying solely on single B-mode US or SE parameters is insufficient for predicting the disease response due to overlapping characteristics between groups [6,7,15]. Combining these parameters has been proven to increase the accuracy of distinguishing between benign and malignant lesions [6,7,19]. In our study, the established US scores comprise a combination of B-mode US parameters, including the S/L ratio, B-mode appearances (contour regularity, margin definition, parenchymal uniformity, presence of nodal hilum, and perinodal fat echogenicity), and SE parameters (ES and B/G ratio). The results demonstrated a significant difference in scores among NLNs, LLNs with partial remission, and LLNs with stable disease. This finding suggests a correlation between the US scores and the response evaluation of the conventional standardized method.

The current study has some limitations. First, ES being a subjective evaluation method, depends on the observer, potentially leading to increased variability among observers. However, this limitation could be addressed using a quantitative measurement such as image color histograms, to enhance in evaluating tissue elasticity [8,31]. Second, this study investigated US parameters of LLNs during the first cycle of chemotherapy due to variations in individual patient conditions, such as chemotherapy side effects, which could hinder treatment completion. To eliminate these confounding factors, we focused on the first chemotherapy cycle. This ensured that all dogs affected with CML received the same chemotherapeutic protocol at a standardized time point. However, as no LLNs were classified as progressive disease or complete response, we were unable to explore differences in US scores between these groups. Further studies should monitor these US parameters throughout the chemotherapy protocol and at tumor relapse. Third, benign lymph nodes such as reactive or lymphadenitis lymph nodes were not included, precluding investigation into differences in US scores between benign and malignant lymph nodes. Nevertheless, previous studies indicate parameters such as the S/L ratio, US appearances, and ES, which were included in our US scores can differentiate between benign and malignant lymph nodes [6,15]. Finally, not all lymph nodes underwent fine-needle aspiration (FNA) to confirm CML diagnosis due to limitations in accessing some lymph nodes located in deeper regions of the body, such as hepatic or splenic lymph nodes. Furthermore, FNA can alter intranodal structure through tissue injury and hemorrhage, leading to tissue repair and the proliferation of fibroblasts and myofibroblasts [37]. This phenomenon could potentially affect the intranodal structure, thus affecting the accurate assessment of US appearances and SE images in our study. To mitigate this, we selected LLNs, that had not undergone FNA based on abnormal ultrasound appearances and size, in accordance with previous studies [6].

## 5. Conclusions

This pilot study revealed significant differences in US parameters, including the B-mode S/L ratio, and SE parameters, including ES and B/G ratio, between NLNs and LLNs. Additionally, these parameters exhibited significant differences between LLN0 and LLN1–LLN3, with no significant differences observed among LLN1–LLN3. In terms of US scores, a significant difference was found between US scores between NLNs, LLNs with partial remission, and LLNs with stable diseases. NLNs showed the lowest US scores, whereas LLNs with stable disease showed the highest US scores. These parameters, in conjunction with the conventional standardized methods, can offer valuable insights into evaluating the treatment response in CML.

## Figures and Tables

**Table 1:** The criteria of ultrasonographic scores.

Criteria	Score
**1. Short to length axis ratio**	
≤0.345	0
>0.345	1
**2. Contour regularity**	
Regular	0
Irregular	1
**3. Margin definition**	
Well-defined margin	0
Ill-defined margin	1
**4. Parenchymal uniformity**	
Homogeneous	0
Heterogeneous	1
**5. Nodal hilum**	
Presence	0
Absence	1
**6. Perinodal fat echogenicity compare with lymph node**	
Isoechoic	0
Hyperechoic	1
**7. Elastrographic scores**	
≤2	0
>2	1
**8. The blue to green ratio**	
<0.745	0
≥0.745	1

**Table 2:** Clinical demographic information of dogs in canine multicentric lymphoma (CML) group and normal group.

Characteristics	CML Group *n* = 15	Normal Group *n* = 15	*p*-Value
Age (years)	9.07 ± 0.74	10.86 ± 0.76	0.102
Gender			<0.001
Male	11 (73.33%)	9 (60%)	
Female	4 (26.67%)	6 (40%)	
Body weight (kg)	9.91 ± 1.39	8.31 ± 2.13	0.536

**Table 3:** The number and frequency of WHO stage and substage, and treatment protocol in canine multicentric lymphoma (CML) group.

Characteristics	CML Group *n* = 15
WHO stage and substage	
Iva	2 (13.33%)
IVb	11 (73.33%)
Va	0 (0%)
Vb	2 (13.34%)
Treatment protocol	
COP	3 (20%)
L-COP	12 (80%)

**Table 4:** The distribution, number, and frequency of lymph nodes of 95 lymphomatous nodes (LLN) and 60 normal lymph nodes (NLN).

Lymph Node	LLN	NLN
Medial iliac lymph nodes	27 (23.28%)	30 (50.00%)
Popliteal lymph nodes	26 (22.41%)	-
Superficial inguinal lymph nodes	26 (22.41%)	30 (50.00%)
Prescapular lymph nodes	16 (13.79%)	-

**Table 5:** Ultrasonographic parameters, including B-mode and strain elastography, of inguinal and medial iliac lymph node, between normal lymph node (NLN) and lymphomatous node (LLN) at the pre-treatment (LLN0), 1 week post treatment (LLN1), 2 weeks post treatment (LLN2), and 3 weeks post treatment.

	NLN (n = 60)	LLN (n = 53)			
		LLN0	LLN1	LLN2	LLN3
B-mode ultrasound					
Short to long axis ratio	0.28 ± 0.01	0.44 ± 0.02 **	0.33 ± 0.02 ^##,^**	0.36 ± 0.03 ^##,^**	0.36 ± 0.02 ^#,^**
Contour regularity					
Regular	60 (100.00)	28 (52.83)	41 (77.36)	43 (81.13)	40 (75.47)
Irregular	0 (0.00)	25 (47.17)	12 (22.64)	6 (11.32)	13 (24.53)
Nodal border definition					
Well-defined	60 (100.00)	50 (94.34)	53 (100.00)	49 (92.45)	53 (100.00)
Ill-defined	0 (0.00)	3 (5.66)	0 (0.00)	0 (0.00)	0 (0.00)
Parenchymal uniformity					
Homogeneous	50 (83.33)	20 (37.74)	34 (64.15)	38 (71.70)	35 (66.04)
Heterogeneous	10 (16.77)	33 (62.26)	19 (35.85)	11 (20.75)	18 (33.96)
Nodal hilum definition					
Present	1 (1.67)	4 (7.55)	4 (7.55)	6 (11.32)	1 (1.89)
Absent	59 (98.33)	49 (92.45)	49 (92.45)	43 (81.13)	52 (98.11)
Perinodal fat echogenicity					
Isoechoic	60 (100.00)	13 (24.53)	33 (62.26)	40 (75.47)	35 (66.04)
Hyperehoic	0 (0.00)	40 (75.47)	20 (37.74)	9 (16.98)	18 (33.96)
Strain elastography					
Elastrographic scales	1 (1–2)	3 (2–4) **	2 (1–3) ^##,^**	2 (1–4) ^##,^**	2 (1–3) ^##,^**
Color histogram					
Red	71.30 ± 2.52	79.49 ± 1.85 **	60.10 ± 2.91 ^#,^*	57.53 ± 3.48 ^#,^**	58.59 ± 3.14 ^#,^**
Green	84.86 ± 2.70	71.68 ± 1.83 **	81.24 ± 2.22 *	81.34 ± 2.61 *	81.74 ± 2.31 *
Blue	56.43 ± 2.59	62.20 ± 1.99	59.94 ± 2.19	61.41 ± 2.30	61.88 ± 2.28
Blue/green ratio	0.66 ± 0.22	0.87 ± 0.23 **	0.74 ± 0.02 ^##,^*	0.79 ± 0.32 ^#,^*	0.76 ± 0.02 ^#,^*

*, significant difference between NLN and LLN0-3 at *p* < 0.05; **, significant difference between NLN and LLN0-3 at *p* < 0.001; ^#^, significant difference between LLN0 and LLN1-3 at *p* < 0.05; ^##^, significant difference between LLN0 and LLN1-3 at *p* < 0.001.

**Table 6:** Ultrasonographic parameters, B-mode, and strain elastography, of a total of 95 lymphomatous nodes (LNNs) at the pre-treatment (LLN0), 1 week post treatment (LLN1), 2 weeks post treatment (LLN2), and 3 weeks post treatment (LLN3).

	LLN0	LLN1	LLN2	LLN3
B-mode ultrasound				
Short to long axis ratio	0.54 ± 0.02	0.44 ± 0.02 *	0.46 ± 0.02 *	0.46 ± 0.02 *
Contour regularity				
Regular	63 (66.32)	81 (85.26)	80 (84.21)	77 (81.05)
Irregular	32 (33.68)	14 (14.74)	7 (15.79)	18 (18.95)
Nodal border definition				
Well-defined	92 (96.84)	94 (98.95)	87 (91.58)	95 (100.00)
Ill-defined	3 (3.16)	1 (1.05)	0 (8.42)	0 (0.00)
Parenchyma uniformity				
Homogeneous	31 (32.63)	45 (47.47)	58 (61.05)	54 (56.84)
Heterogeneous	64 (67.37)	50 (52.63)	29 (38.95)	41 (43.16)
Nodal hilum definition				
Present	7 (7.37)	5 (5.26)	7 (7.37)	3 (3.16)
Absent	88 (92.63)	90 (94.74)	80 (92.63)	92 (96.84)
Perinodal fat echogenicity (n, %)				
Isoechoic	18 (18.95)	47 (49.47)	66 (69.47)	58 (61.05)
Hyperechoic	77 (81.05)	48 (50.53)	21 (30.53)	37 (38.95)
Strain elastography				
Elastrographic score (median, range)	3 (1–4)	2 (1–4) *	2 (1–4) *	2 (1–4) *
Color histogram (mean ± SD)				
Red	41.33 ± 1.53	55.33 ± 2.09 *	53.13 ± 2.28 *	53.65 ± 2.23 *
Green	67.30 ± 1.31	78.11 ± 1.71 *	76.72 ± 1.75 *	78.90 ± 1.71 *
Blue	59.76 ± 1.45	59.96 ± 1.79	58.06 ± 1.74	62.24 ± 1.74
Blue/green ratio	0.89 ± 0.02	0.78 ± 0.03 *	0.78 ± 0.03 *	0.80 ± 0.02 *

*, significant difference between LLN0 and LLN1-3 at *p* < 0.001.

**Table 7:** The cut-off values for ultrasonographic parameters are determined through a receive operating characteristic curve (ROC) and area under curve (AUC) analysis to differentiate between normal lymph node and lymphomatous node.

Ultrasonographic Parameters	Cut-Off	*p*	AUC	Sensitivity	Specificity
Short to long axis ratio	0.345	<0.001	0.876	81.10%	81.70%
Elastographic scale	1.5	<0.001	0.991	100%	93.33%
Blue-to-green ratio	0.745	<0.001	0.815	78.80%	71.70%

## Data Availability

Raw data that supported the finding of this study are available from the corresponding author upon reasonable request.

## References

[1] Vail D.M., Thamm D.H., Liptak J.M. (2019). Hematopoietic Tumors. Withrow and MacEwen’s Small Animal Clinical Oncology.

[2] Montaner-Angoiti E., Marín-García P.J., Llobat L. (2023). Epigenetic Alterations in Canine Malignant Lymphoma: Future and Clinical Outcomes. Animals.

[3] Bennett P., Williamson P., Taylor R. (2023). Review of Canine Lymphoma Treated with Chemotherapy-Outcomes and Prognostic Factors. Vet. Sci..

[4] Vail D.M., Michels G.M., Khanna C., Selting K.A., London C.A. (2010). Response evaluation criteria for peripheral nodal lymphoma in dogs (v1.0)—A Veterinary Cooperative Oncology Group (VCOG) consensus document. Vet. Comp. Oncol..

[5] Alexandrakis I., Tuli R., Ractliffe S.C., Tappin S.W., Foale R.D., Roos A., Slater K.J. (2017). Utility of a multiple serum biomarker test to monitor remission status and relapse in dogs with lymphoma undergoing treatment with chemotherapy. Vet. Comp. Oncol..

[6] Belotta A.F., Gomes M.C., Rocha N.S., Melchert A., Giuffrida R., Silva J.P., Mamprim M.J. (2019). Sonography and sonoelastography in the detection of malignancy in superficial lymph nodes of dogs. J. Vet. Intern. Med..

[7] Febo E., Del Signore F., Bernabò N., Paolini A., Simeoni F., De Bonis A., Rosto M., Canal S., Vignoli M. (2023). Ultrasonography and Sonoelastography Characteristics of Benign vs. Malignant Mesenteric Lymph Nodes in Cats: An Update. Animals.

[8] Choi M., Yoon J., Choi M. (2019). Semi-quantitative strain elastography may facilitate pre-surgical prediction of mandibular lymph nodes malignancy in dogs. J. Vet. Sci..

[9] Silva P., Uscategui R.A.R., Maronezi M.C., Gasser B., Pavan L., Gatto I.R.H., de Almeida V.T., Vicente W.R.R., Feliciano M.A.R. (2018). Ultrasonography for lymph nodes metastasis identification in bitches with mammary neoplasms. Sci. Rep..

[10] Hillaert A., Stock E., Duchateau L., de Rooster H., Devriendt N., Vanderperren K. (2022). B-Mode and Contrast-Enhanced Ultrasonography Aspects of Benign and Malignant Superficial Neoplasms in Dogs: A Preliminary Study. Animals.

[11] Prieto S., Gomez-Ochoa P., De Blas I., Gascón M., Aceña C., Corda A., Sosa I., Gregori T., Couto G. (2009). Pathologic correlation of resistive and pulsatility indices in canine abdominal lymph nodes. Vet. Radiol. Ultrasound.

[12] Salwei R.M., O’Brien R.T., Matheson J.S. (2005). Characterization of lymphomatous lymph nodes in dogs using contrast harmonic and Power Doppler ultrasound. Vet. Radiol. Ultrasound.

[13] Sigrist R.M.S., Liau J., Kaffas A.E., Chammas M.C., Willmann J.K. (2017). Ultrasound Elastography: Review of Techniques and Clinical Applications. Theranostics.

[14] Menzel L., Höpken U.E., Rehm A. (2020). Angiogenesis in Lymph Nodes Is a Critical Regulator of Immune Response and Lymphoma Growth. Front. Immunol..

[15] Seiler G.S., Griffith E. (2018). Comparisons between elastographic stiffness scores for benign versus malignant lymph nodes in dogs and cats. Vet. Radiol. Ultrasound.

[16] Choi Y.J., Lee J.H., Baek J.H. (2015). Ultrasound elastography for evaluation of cervical lymph nodes. Ultrasonography.

[17] Owen L.N., Owen L.N., World Health Organization (1980). Veterinary Public Health Unit & WHO Collaborating Center for Comparative Oncology. TNM Classification of Tumours in Domestic Animals.

[18] Vineela E., Sakalecha A.K., Suresh T.N., Reddy V., Sakalecha A.K., Suresh T. (2022). Role of sonoelastography in differentiating benign from malignant cervical lymph nodes and correlating with pathology. Cureus.

[19] Stan F., Gudea A., Damian A., Gal A.F., Papuc I., Pop A.R., Martonos C. (2020). Ultrasonographic Algorithm for the Assessment of Sentinel Lymph Nodes That Drain the Mammary Carcinomas in Female Dogs. Animals.

[20] Mayer M.N., Lawaon J.A., Silver T.I. (2010). Sonographic characteristics of presumptively normal canine medial iliac and superficial inguinal lymph nodes. Vet. Radiol. Ultrasound.

[21] Davé A.C., Zekas L.J., Auld D.M. (2017). Correlation of cytologic and histopathologic findings with perinodal echogenicity of abdominal lymph nodes in dogs and cats. Vet. Radiol. Ultrasound.

[22] Nyman H.T., Kristensen A.T., Skovgaard I.M., McEvoy F.J. (2005). Characterization of normal and abnormal canine superficial lymph nodes using gray-scale B-mode, color flow mapping, power, and spectral Doppler ultrasonography: A multivariate study. Vet. Radiol. Ultrasound.

[23] Kinns J., Mai W. (2007). Association between malignancy and sonographic heterogeneity in canine and feline abdominal lymph nodes. Vet. Radiol. Ultrasound.

[24] Gödde D., Degener S., Walles C., Keller R., Graf K., Tosch M., Krege S., Musch M., Kvasnicka H.M., Ackermann M. (2023). Degenerative Changes in Aging Human Pelvic Lymph Nodes-A Reason to Rethink Staging and Therapy of Regional Malignancies?. Cancers.

[25] Kwok T., Medovich S.C., Silva-Junior I.A., Brown E.M., Haug J.C., Barrios M.R., Morris K.A., Lancaster J.N. (2022). Age-Associated Changes to Lymph Node Fibroblastic Reticular Cells. Front. Aging.

[26] Nyman H.T., O’Brien R.T. (2007). The Sonographic Evaluation of Lymph Nodes. Clin. Tech. Small Anim. Pract..

[27] Silver T.I., Lawson J.A., Mayer M.N. (2012). Sonographic characteristics of presumptively normal main axillary and superficial cervical lymph nodes in dogs. Am. J. Vet. Res..

[28] Wang B., Guo Q., Wang J.-Y., Yu Y., Yi A.-J., Cui X.-W., Dietrich C.F. (2021). Ultrasound Elastography for the Evaluation of Lymph Nodes. Front. Oncol..

[29] Cè M., Amico N.C., Danesini G.M., Foschini C., Oliva C., Martinenghi C., Cellina M. (2023). Ultrasound Elastography: Basic Principles and Examples of Clinical Applications with Artificial Intelligence—A Review. BioMedInformatics.

[30] Tamura M., Ohta H., Shimbo G., Osuga T., Sasaki N., Morishita K., Kagawa Y., Takiguchi M. (2019). Usefulness of noninvasive shear wave elastography for the assessment of hepatic fibrosis in dogs with hepatic disease. J. Vet. Intern. Med..

[31] Huaijantug S., Yatmark P., Phophug P., Worapakdee M., Phutrakul A., Julapanthong P., Chuaychoo K. (2020). Quantitative ultrasound elastography and serum ferritin level in dogs with liver tumors. J. Adv. Vet. Anim. Res..

[32] Thanaboonnipat C., Sutayatram S., Buranakarl C., Choisunirachon N. (2020). Renal ultrasonographic shear-wave elastography and urinary procollagen type III amino-terminal propeptide in chronic kidney disease dogs. Vet. World.

[33] Massimini M., Gloria A., Romanucci M., Della Salda L., Di Francesco L., Contri A. (2022). Strain and Shear-Wave Elastography and Their Relationship to Histopathological Features of Canine Mammary Nodular Lesions. Vet. Sci..

[34] Brizzi G., Crepaldi P., Roccabianca P., Morabito S., Zini E., Auriemma E., Zanna G. (2021). Strain elastography for the assessment of skin nodules in dogs. Vet. Dermatol..

[35] Secchi V., Masala G., Corda A., Corda F., Potop E., Barbero Fernandez A., Pinna Parpaglia M.L., Sanna Passino E. (2021). Strain Elastography of Injured Equine Superficial Digital Flexor Tendons: A Reliability Study of Manual Measurements. Animals.

[36] Carlsen J.F., Ewertsen C., Săftoiu A., Lönn L., Nielsen M.B. (2014). Accuracy of visual scoring and semi-quantification of ultrasound strain elastography—A phantom study. PLoS ONE.

[37] Chau Y., Chan J. (2003). Fine-needle-aspiration-induced histologic changes. Curr. Diagn. Pathol..

